# Co-located ecological data for exploring top- and subsoil carbon dynamics across grassland-woodland contrasts

**DOI:** 10.1038/s41597-024-03333-w

**Published:** 2024-05-09

**Authors:** Sabine Reinsch, Inma Lebron, Michele Brentegani, Milo Brooks, Susheel Bhanu Busi, Claudia Cagnarini, David Cooper, John Day, Bridget A. Emmett, Eleonora Fitos, Tim Goodall, Robert Griffiths, Briony Jones, Patrick Keenan, Aidan Keith, Josiane M. Lopes-Mazzetto, Kelly E. Mason, Denise Pallett, M. Glória Pereira, Adam Pinder, David A. Robinson, Simon M. Smart, Amy Thomas, Sue Benham, Elena Vanguelova, Bernhard J. Cosby

**Affiliations:** 1 UK Centre for Ecology & Hydrology, Bangor, Gwynedd, LL57 2UW UK; 2 UK Centre for Ecology & Hydrology, Wallingford, OX10 8BB UK; 3 CMCC Foundation - Centro Euro Mediterraneo sui Cambiamenti Climatici, Lecce, 73100 Italy; 4 Bangor University, Bangor, Gwynedd, LL57 2UW UK; 5 UK Centre for Ecology & Hydrology, Lancaster, LA1 4AP UK; 6 Forest Research, Farnham, Surrey, GU10 4LH UK

**Keywords:** Carbon cycle, Biogeochemistry

## Abstract

Soil organic carbon (SOC) is a soil health indicator and understanding dynamics changing SOC stocks will help achieving net zero goals. Here we present four datasets featuring 11,750 data points covering co-located aboveground and below-ground metrics for exploring ecosystem SOC dynamics. Five sites across England with an established land use contrast, grassland and woodland next to each other, were rigorously sampled for aboveground (n = 109), surface (n = 33 soil water release curves), topsoil, and subsoil metrics. Commonly measured soil metrics were analysed in five soil increments for 0–1 metre (n = 4550). Less commonly measured soil metrics which were assumed to change across the soil profile were measured on a subset of samples only (n = 3762). Additionally, we developed a simple method for soil organic matter fractionation using density fractionation which is part of the less common metrics. Finally, soil metrics which may impact SOC dynamics, but with less confidence as to their importance across the soil profile were only measured on topsoil (~5–15 cm = mineral soil) and subsoil (below 50 cm) samples (n = 2567).

## Background & Summary

Soils fulfil multiple functions, food, feed and fibre production, water regulation, gene pool, and mitigating climate change by storing carbon. Understanding and modelling these functions requires new knowledge regarding how soils behave to depth, top and sub-soil. Soil health and capacity to function co-evolves with vegetation, which for example provides carbon inputs that vary with land use. Our understanding of carbon dynamics in soils, which represent the largest terrestrial carbon store^1,2^, is evolving^3^. An opinion paper was published by^4^ exploring the zones of influence of soil organic matter dynamics. The authors present a conceptual framework suggesting that topsoil and subsoil SOC processes are affected by a different set of processes: topsoil processes being primarily driven by land use and climate, and subsoil processes being driven largely by parent material. There is a need for empirical data that enables us to test hypotheses about how carbon is distributed in soils, to depth, and how it persists and the role of land use in building, maintaining, or releasing carbon.

The measure of total SOC is a recognized indicator of soil health^5^: SOC facilitates a living biological habitat enabling SOC-dynamics between the soil physical space and organisms. It is also a key-component of soil health indicator scoring and benchmarking for agricultural practices (e.g.^6,7^. Given the recognition of SOC for soil health and the role a healthy soil plays in achieving national, EU and global net zero goals, the task to accurately predict SOC (stocks) and storage potential under climate change is an important one. In a recent review^8^ assessed ~250 SOC models for their predictive power, showing that the combination of models identifying and evaluating key processes (60% of all SOC models to date) with predictive models will result in better SOC predictions. However, models are only as meaningful as their model structure^9^ and input data^8^. Here we provide unique datasets with co-located aboveground and belowground measurements to facilitate explorative SOC dynamic modelling to 100 cm depth. A wide range of soil chemical, physical and biological metrics were measured including common (e.g. soil pH, SOC) and less common (e.g. soil organic matter fractions, earthworms, and bacterial and fungal metagenomes) soil metrics. We collected the data on a land use contrast featuring long-term (at least 25 years) forest management next to a low-vegetation plot (neutral grassland, bog, fen marsh swamp for at least 10 years) on four soil types. The sampling setup was chosen to disentangle the effects of land use (change) from soil and climatic conditions using predictive modelling approaches.

In 2018, the UK National Environment Research Council funded the “UK Status, Change and Projections of the Environment (UK-SCAPE)” project with the aim to provide national-scale data and models designed to deliver new integrated understanding of the environment. Specifically, significant environmental challenges have been created by pressure on land use, soil quality and biodiversity, which our unique ecological datasets will be used to explore. One part of the programme was set out to 1) better understand the biotic and abiotic controls on the dynamics of SOC, 2) determine where UK soil carbon stocks are most at risk of loss, and 3) identify opportunities to increase SOC storage through land management policies and practices. The empirical data presented will provide new insight into these processes, linking biogeochemical, physical and vegetation metrics.

Recorded aboveground metrics are plant aboveground annual net primary productivity (ANPP) measured at peak growth and litter layer depth (cm). Unsaturated hydraulic conductivity was measured on the soil surface. Soil cores (5 cm deep) were also taken to measure soil water release curves in the laboratory using the HYPROP system. Earthworm abundance and species were measured in the topsoil 25 cm. Commonly measured soil metrics were analysed in five soil increments from topsoil (two increments in the topsoil 0–15 cm) to subsoil (15–30 cm, 30–50/60 cm, below 50/60 cm). Soil metrics considered common were: soil water content (vol/vol), electrical conductivity, bulk density of fine earth, pH in water and CaCl_2_, Loss-on-Ignition (LOI) for soil organic matter (SOM), and total carbon, nitrogen, and phosphorus concentrations. Less commonly measured soil metrics included: Cation Exchange Capacity (CEC), sodium, potassium, calcium, and manganese, extracellular enzyme activities for some of the common enzymes (phosphatase, β-glucosidase, n-acetyl-glucosaminidase, leucine aminopeptidase, phenol oxidase). We also report on enzyme C:N, C:P and N:P ratios and the ratio of simple to complex carbon. Finally, soil metrics only measured on topsoil and subsoil samples were: nitrate-N, dissolved organic carbon (DOC), aggregate size distribution, soil texture (sand/silt/clay), and microbial amplicons and meta-genome-derived abundances were used to derive non-metric multi-dimensional Scaling (nmds) scores for bacterial and fungal communities. A bacterial to fungal ratio is derived from the sequence data. The raw sequence files from the microbial analyses of bacterial and fungal marker genes (16S rRNA gene, and ITS regions respectively) and shotgun whole genome sequencing are deposited in the European Nucleotide Archive (ENA).

## Methods

### Field data collection and laboratory methods

**Fig. 1:**
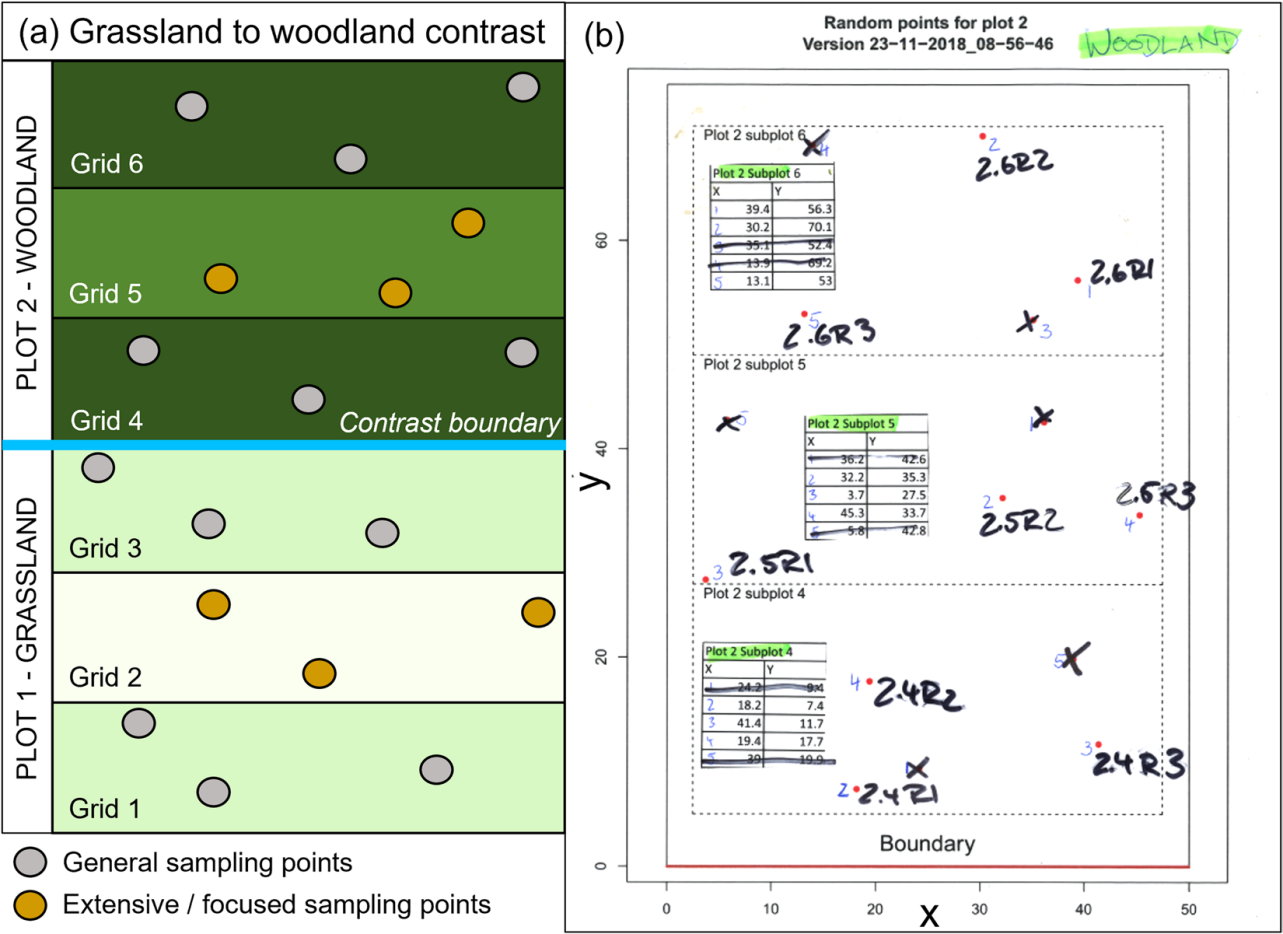
(**a**) Schematic overview of the grassland (Plot 1) to woodland (Plot 2) contrast with the contrast boundary, and grids 1 to 6. Soil sampling was performed in all grids, whereas site specific measurements (ANPP, infiltration, earth worms, HYPROP cores) were only conducted in grids 2 and 5. (**b**) Example of a field map showing the pre-determined randomized points, five of which were provided and three were selected in the field based on e.g. closeness to trees, slope, and other potentially confounding effects.

The impact of land use was regarded as a major anthropogenic driver of SOC dynamics. However, the land use effect is not considered to be independent of soil type and depth. Therefore, sampling locations were chosen where grassland (which for our purposes includes bogs and fen, marsh and swamps) and woodland had been clearly separated for at least 10 years. We avoided arable land and recently felled woodland areas. For all locations, the management history of both land uses had to be available for the last 10 years or longer. The minimum area of each land use was 40 m × 40 m. A gradient sampling strategy was applied to control for unknown soil- and management-derived impacts on measured metrics. Each land use, i.e. grassland and woodland, was divided into three equally sized grids: grasslands grids 1 to 3 and woodland grids 4 to 6 with grids 3 and 4 being the transition zone between grassland and woodland (Fig. 1). A total of 42 locations with 85 grassland-to-woodland contrasts were identified with ten sites approached for access based on soil types common for the UK. Five selected sites were selected (Table 1) and sampled for a variety of aboveground and belowground metrics (Table 2).

**Table 1:** Overview and background information of the chosen sites and management information.

	Gisburn-1	Gisburn-2	Alice Holt	Wytham Woods	Kielder Forest
Location	N England		S England	S England	N England
Decimal latitude / longitude	54.025308, −2.382759	54.026997, −2.383027	51.158516, −0.843573	51.768664, −1.310736	55.209968, −2.468957
Plot size	40 m × 40 m	40 m × 40 m	75 m × 50 m	75 m × 50 m	40 m × 50 m
**MANAGEMENT at time of sampling**					
Grassland	Experiment, un-grazed	Experiment, ploughed, un-grazed	Grazed by cows	Grazed by sheep	Unmanaged
Woodland	Experiment, *Sitka spruce* plantation	Experiment, Mixed deciduous	Fenced, old mixed deciduous	Fenced, grazing excluded	*Sitka spruce* plantation

**Table 2:** Overview of soil and vegetation characteristics and sampling times.

	Gisburn-1	Gisburn-2	Alice Holt	Wytham Woods	Kielder Forest
**SOILS**					
Soils sampled	Oct 2018	Oct 2018	27/28 Nov 2018	Feb 2019	Mar 2019
Infiltration measured	Yes	Yes	No	Yes	Yes for woodland; grassland was water saturated
Earthworms counted	Yes	Yes	No, but data available via Forest Research	No	Yes
Hyprop cores taken	Yes (0–5 cm)	Yes (0–5 cm)	No	Yes (0–5 cm and 30–35 cm)	Yes (0–5 cm)
**VEGETATION**					
Grassland Broad Habitat	Fen, Marsh & Swamp, Bog	Neutral grassland	Neutral grassland	Neutral grassland	Bog
Dominant grassland species	*Juncus acutiflorus*, *Juncus effuses*	*Carex nigra*, *Deschampsia cespitosa*, *Holcus lanatus*	*Alopecurus pratensis*, *Anthoxanthum odoratum*, *Centaurea nigra*, *Trifolium repens*, *Vicia tetrasperma*	*Agrostis stolonifera*, *Lolium perenne*, *Trifolium repens*	*Calluna vulgaris*, *Eriophorum vaginatum*, *Molinia caerulea*
Woodland Broad Habitat	Sitka spruce plantation	Broadleaf woodland	Broadleaf woodland	Broadleaf woodland	Sitka spruce plantation
Dominant woodland species	Young and old *Picea sitchensis*, *Rumex acetosa*	*Alnus glutinosa*, *Holcus mollis*	*Acer campestre*, *Corylus avellana*, *Crataegus monogyna*, *Fagus sylvatica*, *Fraxinus excelsior*, *Hedera helix*, *Mercurialis perennis*, *Quercus robur*	*Corylus avellana*, *Deschampsia cespitosa*, *Mercurialis perennis*, *Quercus robur*, *Rubus fruticosus*	Young and old *Picea sitchensis*
Vegetation sampled	Jul 2021	Jul 2021	May 2021	May 2021	Jul 2021

### Sampling strategy and field methods

A power analysis was performed to estimate the minimum number of samples required to detect a change in soil condition. The ELUM dataset^10^ was used to determine the power to detect SOC change. Bulking soil samples reduces the power to detect potential SOC change due to land use effects. A minimum of three un-bulked soil samples per grid was needed to detect a SOC change of at least 2% between land uses. The power to detect a SOC change of at least 1% would be achieved if there were no confounding factors (e.g. soil texture, deposition gradients, etc.) acting upon the chosen transect. For each site and land use, a field card was produced showing five random points within each grid (Fig. 1b), of which three were chosen in the field based on surrounding conditions (Fig. 1a). Additionally, at Alice Holt and Wytham Woods, the location of the transect was chosen to represent the most uniform area as possible. For Gisburn-1, Gisburn-2 and Kielder Forest, locations were pre-determined by the location of experimental plots and forestry parcels, respectively. Site topography was generally uniform. The only exception was at Wytham Woods where the grassland was located on a slight slope. In this case, the grassland area with the smallest slope was chosen. Coordinates for all sites are provided as detailed in Table 3 if topography is of interest.

**Table 3:** The SOC-D_DATABASE_LOCATIONS.csv datafile holds the location information for all land use contrast sites.

COLUMN_NAME	UNIT	DESCRIPTION
LOCATION_ID	text	Unique ID for each sampling location (xx-yz); xx = site ID (G1, G2, AH, WW, KF); y = grid number (1–6); z = location number (1–3)
GRID_REFERENCE	text	Ordnance Survey Grid Reference (OSGB36) (Ordnance Survey Great Britain)
X	metres	Easting (OSGB)
Y	metres	Northing (OSGB)
LATITUDE	deg	WGS84 Co-Ordinates latitude
LONGITUDE	deg	WGS84 Co-Ordinates longitude

### Plants

Plant species composition was surveyed at each site in grids 2 and 5 within a 2 m by 2 m quadrat at biomass peak times (Table 2). Green leaves were sampled from the two dominant plant species. Leaf dry matter content (LDMC) was determined for these species using dried leaf material. Measured LDMC and LDMC values from databases were used in conjunction with a plant biomass model to estimate annual aboveground net primary production^11^.

### Soils

Before soil sampling started, infiltration measurements were conducted in grids 2 and 5, followed by taking Hyprop soil cores. If sampled during the same campaign, earthworm cores were dug before the 1-m soil sampling started in grid 2. Litter depth was recorded in three locations around the sampling point where litter was present (mainly in the woodlands).

All soil samples were taken after the depth of the litter layer was measured, the soil surface was cleaned of living plant material (mainly grasslands) and then loose litter was removed by brushing away loose leaves (mainly woodlands). All holes were filled afterwards with organic material and soil. The following describes the methods for taking soil cores on different soil types:

- Hyprop cores were taken at 0–5 cm, and if possible, at 30–35 cm depth by hammering the holder (250 mL soil) (Labcell Limited, Hants, UK). The core ends were sealed tightly with clear cling film and plastic caps. Top and bottom was marked. Hyprop cores were used to measure soil water release curves in the labs.
- Deep soil cores (0–100 cm) were taken using different approaches based on the soils. For each core, the depth of the core hole, and the length of the core were documented in the field. This information was necessary to assess potential compaction issues due to the force of the coring equipment (hammering or motor):
  i. Mineral soil cores were either taken fully with a Cobra Precision corer (⌀ 3.9 cm, Van Walt, Ltd, UK) to get a core from 0 to100 cm. If the topsoil was relatively loose and compaction would be high using the Cobra corer, the soil sample was instead taken in two stages: the top 0–30 cm were taken using a Split Tube Sampler with a ⌀ 4.8 cm (Eijkelkamp Soil & Water, Giesbeek, The Netherlands). The Split Tube Sampler has a plastic insert to leave the cores intact. The Cobra precision corer was then used to take the 30–100 cm increment in the same hole. The Cobra corer has flexible plastic liners to keep the core intact. The two parts of the cores were taped together for transport.
  ii. Peat cores or water saturated soils were sampled using a Russian Auger. The length of the corer was 50 cm but only takes half a soil core; cores were taken from 0–50 cm and 50–100 cm to obtain the required depth. Because a Russian Auger has no plastic tube insert, the soil depth increments were cut into sections in the field and bagged in plastic bags. Nitrile gloves were used when samples were extracted from the corer to avoid contamination.

Soil cores were transported to the labs where they were stored at −4°C until processing within 2 weeks. Soils were sectioned into soil depth increments: two depth increments covering the 0–15 cm topsoil (e.g. 0–5 cm and 5–15 cm), 15–30 cm, 30–50 cm or to 60 cm, and the final layer to 100 cm. Soils were combined by sections and subsamples were taken for analyses on fresh soil (e.g. pH and EC). The remaining samples were oven dried at 40°C until dry. Dry soil samples were sieved (2 mm) and quartered before taking subsamples for analyses. Sieved soil sample contained all organic material passing the 2 mm sieve. Dry bulk density was calculated on fine earth i.e. the bulk density after stones were removed using the corer diameter and core section length. Pictures were taken of each core section for reference.

### Common soil metrics

Commonly reported soil metrics were measured on all soil samples and depths. This includes soil water content, electrical conductivity, bulk density, pH in water and CaCl_2_, Loss-on-Ignition (LOI), and total carbon, nitrogen, and phosphorus. The methods are detailed in Table S1.

### Soil metrics measured on a subset of soil cores

Less common soil metrics, which were assumed to change gradually across the soil profile, were measured on samples from grids 2 and 5 only. These included Cation Exchange Capacity (CEC), sodium, potassium, calcium, and manganese. Enzyme activities for some of the common enzyme activities were measured on topsoil (~5–15 cm = mineral soil) and subsoil (below 50 cm) only (phosphatase, β-glucosidase, n-acetyl-glucosaminidase, leucine aminopeptidase, phenol oxidase). We also report on enzyme C:N, C:P and N:P ratios and the ratio of simple to complex carbon. Laboratory methods are detailed in Table S2.

### Soil organic matter fractionation

We present unique data on soil organic matter fractionation; Soil organic matter (SOM) in soil is highly heterogeneous, creating organic matter pools within the soil that may have very different residence times. Models of SOM dynamics must consider these different pools^12^. Conceptual models suggest that stability of SOM depends on the source of plant litter, occlusion within aggregates, incorporation in organo-mineral complexes and location within the soil profile. Density fractionation methodology provides an opportunity to study physical and chemical stabilization mechanisms separating organic debris residing outside (LP-fraction, free light fraction or particulate organic matter, POM) and inside soil aggregates (OP-fraction, occluded particulate light fraction) from mineral-associated organic matter (MA-fraction)^13–16^. A fourth fraction is the dissolved organic carbon (DOC). As its name suggests, this carbon is dissolved in water and therefore may move down in the soil profile or laterally with run-off during big rain events. In the last decade, there has been an increase in DOC concentrations in lakes and streams across Europe and North America attributed to DOC leaching from soils^17^.

**Fig. 2:**
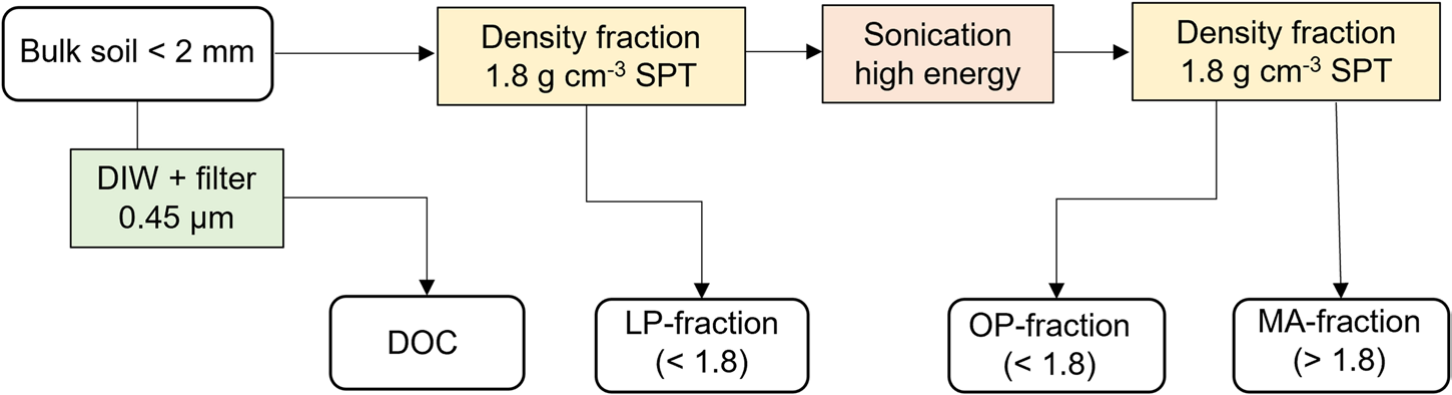
Soil organic matter density fractionation steps starting from sieved bulk soil. DOC = Dissolved Organic Carbon, LP-fraction = Light particulate fraction of soil, OP-fraction = Occluded particulate fraction of soil, and MA-fraction – Mineral-associated fraction of soil. All fractions are expressed in g soil kg soil^−1^ (air dry wt), SPT = Sodium polytungstene.

We designed our fractionation procedure to separate SOM into four discrete fractions of differing stability; these pools are similar to the most frequently used in turnover models: (1) Dissolved organic carbon (DOC), (2) Free light fraction or particulate organic matter (LP-fraction), (3) Occluded particulate (OP-fraction), and (4) OM bound to mineral matter or mineral-associated OM (MA-fraction) (Fig. 2).

### Soil metrics measured on topsoil and subsoil samples only

Metrics which were expected to differ between the topsoil (0–15 cm) and subsoil (below 50 cm) were measured on topsoil and subsoil samples only. Topsoil and subsoil data is available for nitrate-N (NO_3_-N) extracted using ultra-pure water, dissolved organic carbon (DOC), aggregate size distribution (%) (<20 µm, 53 µm, 20–250 µm, 250–2000 µm), texture (sand, silt, clay) and mineral content <30 µm. The instrument measures sizes from 0.04 µm to 2000 µm divided into 116 channels. We consider class 2 µm those measured between 0.4 µm and 2.2 µm and 63 µm those measured between 2.21 and 63.41 µm. For some sites, more measurements were made; for soils from Kielder Forest, the soil material was often too organic and neither aggregates nor texture could be measured.

Microbial amplicon sequencing from eDNA and Metagenomes was performed on topsoil and subsoil samples. The rationale was that the soil microbial community and their functions may considerably vary between topsoil and subsoil. Microbial communities in the intermediate layers were expected to be mixtures of topsoil and subsoil microbial communities. Several metrics were derived from the raw sequence data such as ordination scores (nmds) for bacteria, fungi, and metagenomes respectively, as well as the molecular fungal to bacterial ratios. Methods are described in Table S3.

### Soil hydrological measurements

Soil surface water infiltration rates were measured in the field in grids 2 and 5 *in-situ*. Mini disk infiltrometers were used at different tensions to measure unsaturated hydraulic conductivity between grasslands and woodlands. Data were collected in mL of water infiltrated into the soil over time. Based on the soil type and the tension applied, field data were transferred into a macro (www.decagon.com/macro) to calculate the unsaturated hydraulic conductivity (K). Hydraulic conductivity has been shown to be affected by major land use classes, but Pedotransfer functions used to predict hydraulic conductivity do not (yet) take land use into account^18^.

Hyprop cores (5 cm deep and 250 mL) were taken for the topsoils, and additionally for the mineral soil at Wytham Woods at 30–35 cm depth. Samples were closed at the top and the bottom of the samples using plastic cabs. Soil water release curves were measured in the labs and typically took one month to determine using the evaporation method using a HYPROP system (registered trademark) (UMS, Munich, Germany). The very dry end of the soil water release curves was measured on samples using a humidity chamber to generate two different relative humidities (RH), 40% RH was attained using a saturated solution of potassium carbonate and 80% RH was reached by using a saturated solution of potassium nitrate. Soil aliquots of 1–2 g were dried at 105 °C, weighed to 4 decimal points and placed in the humidity chamber at 40% RH, after 10 d the soils were weighed again to obtain the hygroscopic water retained in the soils at that humidity. The same procedure was repeated for the 80% RH. These two points, at 40% and 80% RH, correspond to ~30 MPa and 124 MPa and were used to direct the dry end of the water release curves. Soil water release curves can be fitted to the data^19^ and allow the evaluation of soil hydrological conditions across soil types and management options^20^. The effective pore size distribution may be derived from the data giving some insight into the soil structural arrangement. This will give insight into the potential of the soils to store water.

### Earthworms

In the field (grids 2 and 5), vegetation was clipped to ground level with hand-shears and a 25 cm × 25 cm square soil block was excavated to a depth of 25 cm. The soil block was placed on a plastic sheet and sorted through by hand to collect the earthworms. Hand-sorting was standardized by limiting sorting time to 15 min. Collected earthworms were placed in plastic bottles in the field and these were kept in cool-boxes containing icepacks. In the laboratory the earthworms were kept cool (4°C) until they were weighed and identified to species level.

## Data Records

Four datasets are available through the Environment Information Data Centre (EIDC) and are part of the data collection “Co-located ecological data from five long-term grassland-to-woodland land use contrasts across England measured between 2018 and 2021” (https://catalogue.ceh.ac.uk/documents/b8081717-5ba9-48f8-a7f4-aef49642b4ef).

Each dataset contains a supporting document detailing the data formats and data structure. Additionally, two data files are provided to each of the four environmental datasets which can be used to combine the co-located measurements: The SOC-D_DATABASE_LOCATIONS.csv (Table 3) includes site IDs and coordinates. The SOC-D_DATABASE_CONNECTOR.csv (Table 4) includes sample IDs which can be used to connect the co-located measurements. Missing values in the datasets are NAs.

**Table 4:** The SOC-D_DATABASE_CONNECTOR.csv provides information on the experimental layout.

COLUMN_NAME	UNIT	DESCRIPTION
SITE_NAME	text	Site name
LANDUSE	text	Grassland or Woodland part of the land use contrast
SITE	integer	Site (1–5) – Number for each site
PLOT	integer	Plot (1–2) – Number for area sampled on either side of land use contrast; Plot 1 = grassland, Plot 2 = woodland
GRID	integer	Grid (1–6) – Rectangular areas parallel to and at stratified distances from the woodland/grassland land use contrast; Grids 1 to 3 are in grassland; Grids 4 to 6 are in woodland (3 and 4 are adjacent to the transition)
CORE	integer	Core (1–3) – Individual cores taken within each grid; core locations randomly selected within grids
TRANSITION_DISTANCE_m	m	Distance of core location from transition boundary (Y axis: Positive upward for woodland cores, Negative downward for grassland cores)
LATERAL_DISTANCE_m	m	Distance of core location from grid edge (X axis: Positive to the right for both woodland and grassland cores)
SLICE	integer	Slice (1,2, … n) – Number for each layer/section/slice extracted from a core for analysis (surface = 1)
LAB_LAYER_cm	text	Layer description – Distance of top & bottom of core slice from top of core; measured when processing cores in lab; note the space before the increment
LAYER_DEPTH_TOP_cm	cm	Depth from surface of soil at top of core slice
LAYER_DEPTH_BOTTOM_cm	cm	Depth from surface of soil at bottom of core slice
LOCATION_ID	text	Unique ID for each sampling location (xx-yz); xx = site ID (G1, G2, AH, WW, KF); y = grid number (1–6); z = location number (1–3)
SAMPLE_ID	text	Unique ID for each sample taken from soil cores (CoreID-Layer)

### Plants

The dataset “Plant aboveground net primary productivity estimates (2021) and litter layer depth measurements (2018–2019) at five long-term grassland-to-woodland land use contrasts across England”^21^ contains two environmental data files, one holding ANPP estimates and the other file containing the litter layer depth measurements.

### Soils

The dataset “Soil physical, chemical, and biological properties (0–1 m) at five long-term grassland-to-woodland land use contrasts across England, 2018–2019”^22^ contains three environmental data files holding soil variables measured at different spatial resolutions. 1) Soil metrics in the file SOC-D_DATABASE_COMMON_SOIL_METRICS.csv were measured for all sampling locations and all depths. 2) The SOC-D_DATABASE_ SOIL_METRICS_GRID2_GRID5.csv datafile contains metrics measured in grid 2 (grassland) and grid 5 (woodland) across all depths. 3) Soil metrics in the SOC-D_DATABASE_SOIL_METRICS_TOP_AND_SUB_SOIL.csv datafile were measured for topsoil (0–15 cm) and subsoil (below 50 cm) only, but for all grids (1–6).

### Soil hydrological measurements

The dataset “Soil water release curves and hydraulic conductivity measurements at four long-term grassland-to-woodland land use contrasts across England”^19^ contains four environmental data files which include the soil hydraulic conductivity data (SOC_D_DATABASE_SOIL_HYDRAULIC_ CONDUCTIVITY.csv) and the HYPROP raw data. The latter three datasets are the output of the HYPROP system detailing pF value and volumetric water content, pF and log_10_ hydraulic conductivity, and volumetric water content and log_10_ hydraulic conductivity.

### Earthworms

The dataset “Earthworm species identification and counts at three long-term grassland-to-woodland land use contrasts across England”^23^ contains the datafile SOC_D_DATABASE_EARTHWORMS.csv. Earthworm counts for a range of species are reported as well as their weights.

### Molecular raw sequencing files

Raw sequence files from the microbial analyses of bacterial and fungal marker genes (16S rRNA gene, and ITS regions respectively) and shotgun whole genome sequencing are deposited in the European Nucleotide Archive (ENA) under project accession PRJEB66294 (https://identifiers.org/ena.embl:PRJEB66294 (2023))^24^.

## Technical Validation

Location data were visually checked by BJC using field sheets, GPS coordinates, and google Earth maps. All data derived from UKCEH laboratories were visually checked for their accuracy in comparison to other metrics available, and re-measured where required.

UKCEH maintains a quality management system across its four sites which is ISO 9001:2015 certified. The laboratories at the UKCEH Lancaster are UKAS (United Kingdom Accreditation Service; https://www.ukas.com/, last day issued: 11 May 2023) accredited. Soils were analysed at UKCEH Lancaster for total carbon, total nitrogen and total phosphorus concentrations.

Soil samples were processed at the UKCEH Bangor laboratory where yearly more than 1000 soil cores are analysed for national scale (soil) surveys^25–28^. A robust protocol is in place for processing soil cores for bulk density of fine earth, pH (in water and CaCl_2_), electric conductivity, and Loss-on-Ignition determined from thermos-gravimetric analysis (TGA)^29^. Two internal long-term standards (BS1 and BS3), both Brown Earth from the UK (Wales), are used in each batch as well as 10% of replicated samples to account for accuracy and precision. Values for both standards need to be within 2 standard deviations of the long-term mean to pass the quality.

UKCEH Bangor laboratory also subscribes to the WEPAL scheme (https://www.wepal.nl/en/wepal/about-us.htm) which is a proficiency test that externally evaluates the accuracy of particle size distribution measurements.

Specific information on quality control and precision measures is documented with each method described in the three Tables S1–S3.

### Supplementary information

Supplementary information - revised

## Data Availability

ANPP was estimated using a published method by Smart *et al*.^11^ using the BUGS model as described therein. For amplicon datasets, reads were paired, quality checked and clustered into operational taxonomic units (OTUs) using the DADA2 pipeline using default settings https://benjjneb.github.io/dada2/. To derive a metric of the ratio of fungi to bacteria, pre-processed metagenomic reads were taxonomically annotated using the Kraken2 software https://github.com/DerrickWood/kraken2 and the PlusPFP reference database containing genomes from all domains of life https://benlangmead.github.io/aws-indexes/k2.
