# Supplementary material for: Co-located ecological data for exploring top- and subsoil carbon dynamics across grassland-woodland contrasts
Source: Sci Data. 2024 May 9;11:478. doi: 10.1038/s41597-024-03333-w (PMC11081944; doi:10.1038/s41597-024-03333-w)
Supplement: Supplementary file 1 — Supplementary information - revised [file 41597_2024_3333_MOESM1_ESM.docx]

## Co-located ecological data for exploring top- and subsoil carbon dynamics across grassland-to-woodland contrasts

## Supplementary Information

Reinsch S, Lebron I et al.

**Table of contents**

**Table S1**: Summary of laboratory methods for commonly reported soil metrics.

**Table S2**: Laboratory method descriptions for metrics measured across the depths profile in grids 2 and 5.

**Table S3**: Laboratory method descriptions for metrics measured on topsoil and subsoil layers.

**Supplementary tables**

**Table S1: Summary of laboratory methods for commonly reported soil metrics**.

| Method details | References |
| --- | --- |
| Soil Electrical Conductivity (EC, µS cm^-1^) |  |
| EC was measured on a suspension of 10 g of fresh field-moist soil with 25 mL of deionised water, the suspension is stirred with a rod to produce a homogeneous suspension. After half an hour, the content of the beaker was stirred again with the rod, and the EC was measured using an electrode and a conductivity meter (Jenway 4510). The instrument was calibrated with KCl standards of known EC before every batch was measured. For quality control, two different internal standards with known EC values were included in each batch; we also include duplicates in our batch (about 10% of the total number of samples). The internal standards assure accuracy in our measurements while the repeats ensure precision. |  |
| Soil pH in deionized water (DIW) and calcium chloride (CaCl_2_) |  |
| Soil pH in DIW was measured on the same beakers after measuring the EC. After measuring pH in DIW, 2 mL of 0.125 M CaCl_2_ was added to the beakers to achieve a total concentration of 0.01 M CaCl_2_. Suspensions were stirred and allowed standing for 15 min after which the pH was measured again; this value is the reported as pH in CaCl_2_. The instrument was calibrated before every batch with two buffer standards, pH 4 and pH 7. The same two internal standards and repeats used for EC as well as 10% of replicated samples provide the quality control for accuracy and precision. | ^1^ |
| Bulk density of fine earth (g cm^-3^) |  |
| Bulk density is the expressed as the amount of dry fine earth (sieved to 2 mm) per unit volume. |  |
| Loss-on-Ignition (LOI, g 100 g^-1^ soil, %) |  |
| LOI was determined using thermogravimetric analysis (TGA) using a LECO TGA 701 model. TGA is a simple and inexpensive method for determining soil organic matter and estimating soil organic carbon concentration using an appropriate conversion equation that can be determined using total carbon analysis. The TGA records weights automatically at each time step. LOI was measured on a 10 g air dried sub-sample taken after sieving to 2 mm. The temperature was increased rapidly from 25°C to 105°C at a rate of 6°C per minute and allowed the sample for 3.5 h to remove moisture. The temperature was increased at the same rate of 6°C per minute to reach 375°C and allowed at that temperature for 16 h. The weight loss between 105°C at 375°C is LOI. LOI quality control checks were carried out using internal soil standards prepared in an identical manner to the soils. Two different internal standards were included in each sample batch as well as 10% of replicated samples to account for accuracy and precision. Those internal standards were compared with a historically generated mean value. If the measured LOI for the two internal standards in a batch varied by more than 2 standard deviations, in either direction, from the historic mean value, then the batch was repeated. The TGA is under maintenance contract, microbalance and oven temperatures are calibrated once per year. | ^2^ |
| Total carbon and nitrogen (%) |  |
| Total carbon and total nitrogen were analysed for bulk soil, and organic carbon fractions. Soil samples were air dried, ball milled, and oven dried at 105°C (± 5°C) for a minimum of 3 h. Samples (10-20 mg) were then analysed using an Elementar Vario-EL elemental analyser (Elementaranalysensysteme GmbH, Hanau, Germany). This analysis was carried out using the UKAS accredited to 17025 standard. Quality control was achieved by use of two in-house reference materials analysed with each batch of samples. | ^3^ |
| Total phosphorus (mg P kg^-1^ soil) |  |
| A subsample of 0.3-0.4 g air-dried soil (sieved through 2 mm) was digested with hydrogen peroxide (100 Volumes) and concentrated sulphuric acid in a 5:6 ratio. Selenium powder and lithium sulphate were added to raise the boiling point of the acid. Samples were then digested at 250^o^C for 15 min and then to 400^o^C for 2 h. After digestion, the samples were diluted with ultrapure water and allowed to settle overnight. The supernatant was then further diluted, and phosporus was measured by using ammonium molybdenum blue chemistry with the addition of ascorbic acid to control the colour production, a SEAL AQ2 was the discrete analyser used to quantify concentrations. Two quality control reference samples, a duplicate sample and two matrix matched blanks were run every 25 samples to ensure data quality. The final concentration was determined using a calibration curve of the standard and took into account the blank concentration. |  |

**Table S2: Laboratory method descriptions for metrics measured across the depths profile in grids 2 and 5.**

| Method details | References |
| --- | --- |
| Exchangeable cations (mg kg^-1^ soil) and cation exchange capacity (meq 100 g^-1^) |  |
| A subsample of 2 g of air-dried soil was extracted with 1 M ammonia acetate (pH 7.0) and shaken on a rotary end-over-end shaker for 1 h. The extracts were filtered through ashless Whatmann 44 filter paper prewashed with distilled water. The filtrate was stored in the fridge at 4^o^C for (exchangeable) cation analysis by ICP-OES. Calcium, magnesium, potassium, and sodium were measured using a Perkin Elmer model 7300DV Inductively Coupled Plasma-Optical Emission Spectrometer (ICP-OES, Perkin Elmer, Waltham, USA). The elements were quantified using matrix matched standards calibration curves. For quality control purposes, blanks, an Initial Calibration Verification Standard (ICVS: Perkin Elmer) and 2 certified reference materials were routinely scheduled throughout the instrument batch analyses alongside repeat samples.  In the second stage, the residue on each filter paper was washed once with a combined solution of 4:1 60% IMS: 10% w/v ammonium chloride followed by, at minimum, 3 times wash with 20 mL of 60% IMS; the filtrate was discarded. The remaining residue on the filter was finally washed again with 60% IMS and the resulting filtrate was tested for unbound NH_4_Cl using 3 drops AgNO_3_ (white precipitate formed in the presence of chloride). If precipitate was formed, it would be further washed with 60% IMS until there was no unbound NH_4_Cl in the filtrate. Once the filtrate was free of NH_4_Cl, 5% KCl was passed through the filter to displace the NH_4_, the filtrate collected was analysed for total ammoniac nitrogen, i.e. ammonium nitrogen (NH_4_-N) and ammonia nitrogen (NH_3_-N), measured colourimetrically using a SEAL AA3 continuous flow analyser (SEAL Analytical, Germany). The reaction was carried out under alkaline conditions, which ensured that any ammonium ions were converted to ammonia and measured in the following reaction. A calibration range of 0-10.0 mg L^-1^ NH_3_-N was used, and concentrations were obtained using the calibration curve. Certified reference materials containing NH_4_-N were used to check measurement accuracy. |  |
| Extracellular enzyme activity (µmol substrate g^-1^ soil h^-1^) |  |
| Hydrolytic enzymes acquiring cellulose-C (β-glucosidase), chitin-N (N-acetylglucosaminidase), leucine-N (leucine amino peptidase), and phosphorous (phosphatase) were assayed using substrates tagged with fluorescing 4-methylumbelliferone (MUB), or 7-amino-4-methyl coumarin (MC) for leucine amino peptidase. The oxidative enzyme acquiring phenol-C (phenol oxidase) was assayed spectrophotometrically. Prior to analysis, samples were stored at -20°C. Soil samples (1 g) were suspended in 125 mL buffer using a laboratory-grade blender. A modified universal buffer (pH 7) was used for neutral soils and a sodium acetate buffer (pH 5) was used for acidic soils. For hydrolytic enzyme activities, soil suspensions (100 µL, 8 replicates) were pipetted into 96-well plates and 100 µL of target substrate added. Quench standards (MUB or MC) and buffer blanks were included for each replicate. Plates were incubated in the dark at 15°C, and fluorescence measured repeatedly over 2 h using a BioTek Cytation 5 Cell Imaging Multimode Reader (Agilent Technologies, Inc., Santa Clara, USA) with 360 nm excitation and 460 nm emission filters. For phenol oxidase activity, soil suspensions (600 µL, 8 replicates) and 400 µL 3,4-Dihydroxy-L-phenylalanine (L-DOPA) substrate were pipetted into deep 96-well plates. Sample, background (soil suspension + buffer), and L-DOPA standard plates were incubated in the dark at 15°C and absorption measured at 460 nm after 24 h, also on the Biotek Cytation 5 Multimode Reader. Enzyme activity rates were corrected for blanks and standards. | ^4^ |
| Organic matter fractionation (g fraction kg^-1^ soil) – Figure 2 |  |
| Quantification of dissolved organic matter (DOC) was performed on 2.5 g of air-dry 2 mm sieved soil weighed in 50 mL falcon tubes with 25 mL of ultrapure water. Tubes were shaken for 1 h at 200 rpm. Visual observation is required to assure that the soil is completely wet; soils displaying hydrophobicity have the tendency to remain on the surface of the water and may need more vigorous stirring to assure total water immersion. Tubes were centrifuged for 5 min at 3935 RFC, the supernatant was decanted and filtered through a pre-rinsed 0.45 µm filter. Non-purgeable organic carbon (NPOC) and total nitrogen (TN) were analysed in the supernatant using a Thermalox instrument (Analytical Sciences, UK).  On a new soil aliquot, 40 mL of 1.8 g cm^-3^ sodium polytungstate (SPT) was added to 2.5 g of air-dried and sieved (2 mm) soil in a 50 mL falcon tube. Tubes were shaken for 30 min at 100 rpm, again special attention needs to be taken when soils show hydrophobicity. Centrifuge at 1874 rfc for 30 min. The supernatant with the light fraction of the organic matter was then filtered carefully to not disturb the pellet at the bottom of the tube into a filtering vacuum device with polyethersulfone 0.45 µm filters. Material was rinsed with deionised water (DW) onto the filter until the electrical conductivity of the outcome filtered liquid was below 100 µS cm^-1^, except for calcareous soils where 200 µS cm^-1^ is acceptable. The material on the filter was then transferred to a pre-weighed pre-labelled aluminium boat using DW; the boat was placed in the oven at 105°C to evaporate the water. The final weight of the boat when dry was recorded to obtain the weight of the LP-fraction.  The remaining pellet in the falcon tube was re-suspended in another aliquot of 40 mL of 1.8 g cm^-3^ SPT using a vortex instrument. The tube was placed in an ice bath to keep the temperature of the suspension below 40°C and was submitted to ultrasonic sonication to break down the soil aggregates. The amplitude was fixed to 60% in the ultrasonic instrument and sonicated for 5 min to obtain a minimum of 50 W. After sonication the procedure described above was repeated: tubes were centrifuged, supernatant filtered, rinsed, and collected. The weight of the second boat provides the occluded particulate fraction (OP-fraction).  The residual material in the falcon tube is the mineral-associated organic matter (MA-fraction). To remove the SPT from the soil 40 mL of DW was added, and the tubes centrifuge at 3935 RFC for 0.5 h. This step was repeated at least 4 times until the electrical conductivity of the supernatant was below 100 µS cm^-1^. As before, material was transferred into a pre-weighed aluminium boat and weighed after drying at 400°C to obtain the MA-fraction. Two internal standards were used in each batch as well as 10% of replicated samples to account for accuracy and precision. | ^5–7^ |

**Table S3: Laboratory method descriptions for metrics measured on topsoil and subsoil layers.**

| Method details | References |
| --- | --- |
| Nitrate-N (mg N g^-1^ soil) |  |
| Approx 2.5 g field moist soil was weighed into 50 mL centrifuge tubes, 25 mL of ultrapure water was added, and the vials were shaken horizontally for 1 h at 200 rpm. Shaken samples were centrifuged for 5 min at 4000 rpm, before being filtered using AcroCap SuPor 0.45 µm filters, that were pre-washed with 150 mL ultrapure water, with the first 5 mL of filtrate being discarded. Samples were analysed on a SEAL AQ2+ discrete analyser, fitted with a cadmium reduction coil, using the sulfanilamide - N.E.D.D. method in EPA-126-A Rev. 4. Absorbance was measured at 660 nm within 48 h of extraction. |  |
| Dissolved organic carbon (mg C g^-1^ soil) |  |
| A subsample of 2.5 g of field moist soil was weighed and the mass of soil was recorded to the nearest 0.01 g. After adding 25 mL of ultrapure water the samples, including two blanks, samples were shaken for 1 h at 200 rpm. Samples were then centrifuged for 5 min at 4000 rpm and the supernatant was filtered through pre-washed blue AcroCap Supor 0.45 μm filters. Samples were stored at 4^o^C until analysis. Total carbon and inorganic carbon were analysed in the filtered supernatants in a Thermalox instrument. |  |
| Particle size distribution (PSD) and soil texture classes (% sand, % silt, % clay) |  |
| A soil subsample of ~0.5 g the soil was placed in a digester to remove the organic matter (OM). The heating digester unit was a VELP scientifica (DK) with a capacity of 42 tubes in total. Test tubes (26 mm diameter by 300 mm long) were used in the digester. 6% hydrogen peroxide was used in the first instance to oxidize the more reactive organic carbon and subsequently 30% hydrogen peroxide was used combined with three heating cycles of 85^o^C, 100^o^C and 110^o^C for 1 h each. When the full digest cycle was complete, the sample should have a clear supernatant with sand/particles at bottom. The sample was transferred into 250 mL bottles, 5 mL of 5% Calgon was added. Samples were placed it in an orbital shaker overnight. The content of the 250 mL bottles was emptied manually into the particle size analyser for the measurement of PSD. Sonication of 50 W for 6 s was applied to the samples to ensure total breakdown of aggregates before proceeding to the measurement.  Laser diffraction (LD) was used to analyse the soils for PSD. LD has the advantage of requiring smaller amounts of soil (<2.0 g) than the traditional hydrometer method, is very reproducible and provides a wide range of size classes (rather than the conventional 3 to 9). The instrument used was a Beckman Coulter LS13 320 laser diffraction particle size analyser (Beckman Coulter Inc). Standard soil samples were included with each batch of samples and duplicated samples were included (one in ten) to check for reproducibility. The sand fraction was collected with a 63 μm sieve at the end of the drainage outlet. To corroborate the laser measurements, the weight of the sand collected in the sieve at the end of the measurement was compared with the data provided by the instrument. There was a good agreement for both values for the sand fraction. Two internal standards with known particle size distribution were used in each batch as well as 10% of replicated samples to account for accuracy and precision, the instrument is calibrated and maintained yearly to comply with the ISO 9001 regulations. We also subscribe to the WEPAL scheme (https://www.wepal.nl/en/wepal/about-us.htm) which is a proficiency test that externally evaluates the accuracy of our particle size determinations. We reported the clay, silt, and sand fractions as those <2 µm, 2-63, and >63 µm. | ^8^ |
| Aggregate size distribution (ASD, µm) |  |
| ASD was measured using the laser particle analyser as described above for PSD. For ASD, organic carbon was not removed. A soil subsample of aggregates smaller than 2 mm but bigger than 1 mm was introduced into the laser analyser, the amount of soil necessary for this analysis was that to achieve at least 1-2% in the obscuration parameter in the LD, this minimum is required for the software to collect enough scattering patterns to produce a reproducible result. The data were reported in three aggregate sizes according to the models in the literature to describe soil aggregation. The lowest hierarchical order is the micro-aggregates (less than 20 µm) consisting of clay particles bonded by physico-chemical forces and attached to organic molecules by polyvalent cations, the next hierarchical order is the combination of these micro-aggregates into bigger aggregates or meso-aggregates (> than 20 µm but <250 µm). These meso-aggregates are held together by a number of cementing agents, e.g., sesquioxides or carbonates, it has also been shown that encrustation of plant debris is an integral part of the stabilisation of these aggregates. The next hierarchical order is the combination of meso-aggregates <250 into macro-aggregates >250 µm held together by fine roots and fungal hyphae, this size class has also been shown to enclose particulate organic matter within the aggregates. Two internal standards with known aggregate size distribution were used in each batch as well as 10% of replicated samples to account for accuracy and precision. The instrument is calibrated and maintained yearly to comply with the ISO 9001 regulations. | ^9–12^ |
| Soil microbial DNA for amplicon and metagenome analysis |  |
| Soil community DNA was extracted from 0.2 g of field moist soil using the PowerSoil HTP DNA isolation kit (Qiagen), and 2.5 g field moist soil using the PowerMax Soil DNA kit (Qiagen), for amplicon sequencing and metagenome analysis respectively and following manufacturer’s protocol. Samples for metagenome analysis were concentrated using Amicon-Ultra 100kDa MWCO to achieve sufficient concentration for sequencing. Extracted DNA was quantified, and quality checked by fluorometer Qubit® 2.0 dsDNA BR Assay Kit (Thermo Fisher Scientific) and Nanodrop (Thermo Fisher Scientific), following manufacturer’s protocol.  Prokaryotic and fungal community composition were assessed by amplicon sequencing the V4-V5 region of the 16S rRNA genes using the 515f GTGYCAGCMGCCGCGGTAA and 806r GGACTACNVGGGTWTCTAAT primers and the established primers GTGARTCATCGAATCTTTG and TCCTCCGCTTATTGATATGC coding the ITS2 region respectively, following the PCR protocols of the Earth Microbiome Project. Sequencing was performed using a 2-step Nextera approach on the Illumina MiSeq platform using V3 chemistry (Illumina Inc., USA). For both amplicon datasets, reads were paired, quality checked and clustered into operational taxonomic units (OTUs) using the DADA2 pipeline using default settings https://benjjneb.github.io/dada2/.  Metagenome sequences were produced using a PCR-shotgun library kit on Novoseq 6000. Illumina adaptor sequences were removed using cutadapt 4.1, reads were then trimmed with Sickle 1.33 with a minimum window quality score of 20. Reads shorter than 20bp after trimming were discarded. Forward reads were functionally annotated using DIAMOND BLASTX v0.8.38.100 matching against the SAMSA2 SEED subsystems database.  Tables of counts of OTUs or genes were then resampled to equivalent read depth prior to ordination using the metaMDS function within the R vegan library. Sample metrics corresponding to the first and second axis of variation are available along with assayed concentration of nucleic acid extracted, which has in the past been used as a microbial biomass proxy. Finally, to derive a metric of the ratio of fungi to bacteria, pre-processed metagenomic reads were taxonomically annotated using the Kraken2 software https://github.com/DerrickWood/kraken2 and the PlusPFP reference database containing genomes from all domains of life <https://benlangmead.github.io/aws-indexes/k2>. | earthmicrobiome.org  ^13–15^ |

**References**

1. Avery, B. & Bascomb, C. Soil Survey Laboratory Methods. *Soil Survey Technical Monograph* No. 6 (1974).

2. Lebron, I. *et al.* Soil organic matter determination for long-term monitoring revisited using thermogravimetric analysis. *Vadose Zone Journal* (2023).

3. Emmett, B. A. *et al.* *Countryside Survey: Soils Report from 2007*. http://nora.nerc.ac.uk/9354/1/CS_UK_2007_TR9.pdf (2010).

4. Saiya-Cork, K. R., Sinsabaugh, R. L. & Zak, D. R. The effects of long term nitrogen deposition on extracellular enzyme activity in an Acer saccharum forest soil. *Soil Biol Biochem* **34**, 1309–1315 (2002).

5. Schrumpf, M. *et al.* Storage and stability of organic carbon in soils as related to depth, occlusion within aggregates, and attachment to minerals. *Biogeosciences* **10**, 1675–1691 (2013).

6. Poeplau, C. & Don, A. Effect of ultrasonic power on soil organic carbon fractions. *Journal of Plant Nutrition and Soil Science* **177**, 137–140 (2014).

7. Cotrufo, M. F. *et al.* Formation of soil organic matter via biochemical and physical pathways of litter mass loss. *Nat Geosci* **8**, 776–779 (2015).

8. Lebron, I. *et al.* Topsoil particle size distribution from the Glastir Monitoring and Evaluation Programme, Wales 2013-2016. *NERC Environmental Information Data Centre (Dataset)* Preprint at https://doi.org/https://doi.org/10.5285/d6c3cc3c-a7b7-48b2-9e61-d07454639656 (2020).

9. Oades, J. M. & Waters, A. G. Aggregate hierarchy in soils. *Soil Research* **29**, 815–828 (1991).

10. Golchin, A., Baldock, J. A. & Oades, J. M. A model linking organic matter decomposition, chemistry, and aggregate dynamics. in *Soil processes and the carbon cycle* 22 (CRC Press, 1997).

11. Edwards, A. P. & Bremner, J. M. Microaggregates in soils. *Journal of Soil Science* **18**, 64–73 (1967).

12. Tisdall, J. M. & Oades, J. M. Organic matter and water-stable aggregates in soils. *Journal of Soil Science* **33**, 141–163 (1982).

13. Ihrmark, K. *et al.* New primers to amplify the fungal ITS2 region – evaluation by 454-sequencing of artificial and natural communities. *FEMS Microbiol Ecol* **82**, 666–677 (2012).

14. Walters, W. *et al.* Improved Bacterial 16S rRNA Gene (V4 and V4-5) and Fungal Internal Transcribed Spacer Marker Gene Primers for Microbial Community Surveys. *mSystems* **1**, 10.1128/msystems.00009-15 (2016).

15. Callahan, B. J. *et al.* DADA2: High-resolution sample inference from Illumina amplicon data. *Nat Methods* **13**, 581–583 (2016).

16. ENA European Nucleotide Archive. <https://identifiers.org/ena.embl:PRJEB66294> (2023).
